# Ipsilateral Acetabular Fracture with Displaced Femoral Head and Femoral Shaft Fracture: A Complex Floating Hip Injury

**DOI:** 10.1155/2018/4937472

**Published:** 2018-07-03

**Authors:** Raja Bhaskara Rajasekaran, Dheenadhayalan Jayaramaraju, Dhanasekara Raja Palanisami, Ramesh Perumal, Rajasekaran Shanmuganathan

**Affiliations:** Department of Orthopaedics & Trauma, 313 Mettupalayam Road, Ganga Medical Centre & Hospitals Pvt. Ltd., Coimbatore, India

## Abstract

Floating hip injuries involving the acetabulum, femoral head, and the femoral shaft are a very rare presentation. A complex floating hip injury comprising of an ipsilateral acetabular fracture associated with a displaced femoral head fracture and a femoral shaft fracture following a high-velocity road traffic accident presented to us where all the fractures were addressed with internal fixation during the primary surgery. Postoperatively, the patient suffered a dislocation of the femoral head which eventually went on to avascular necrosis at 5 months from the initial presentation. Then, the patient underwent a total hip replacement with an acetabular reconstruction following which he went on to have a good functional outcome. Our experience in dealing with such a complex case shows that it is difficult to set a protocol for such injuries and they need to be addressed on a case-to-case basis depending on the complexity of the injury.

## 1. Introduction

A “floating hip” injury—a fracture of the pelvis or acetabulum with a concomitant fracture of the femur—is a very rare presentation with an incidence of about 1 in 10,000 fractures [1–3]. The combination of an ipsilateral acetabular fracture with a displaced femoral head fracture and a femoral shaft fracture is an even rarer presentation. Only two cases of such injuries have been described so far and there is paucity in literature regarding the management of such cases [4]. Here, we present such a case following a road traffic accident and the challenges we faced while addressing all the fractures.

## 2. Case Report

A 52-year-old farmer was referred to us 7 hours after he had met with a high-velocity road traffic accident. He was resuscitated as per the ATLS protocol at the hospital where he was initially treated and when he arrived at the casualty department of our hospital, he was conscious and all his vital parameters were within normal limits. He also gave the history that he was under treatment for segmental myoclonus which was characterised by semirhythmic involuntary muscle contractions.

**Figure 1:**
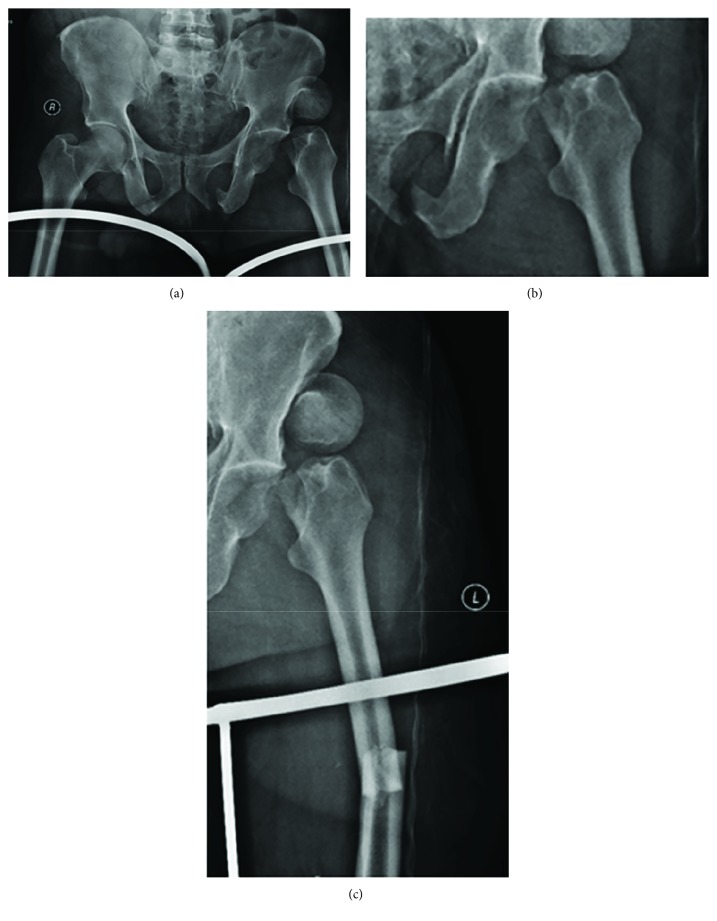
Radiographs of the pelvis (a), showing a posterior wall acetabulum fracture (b) with a displaced femoral head and a concomitant femur shaft fracture (c).

His radiographs showed a left-sided posterior acetabular wall fracture (AO type 6 2 A1). He also had an ipsilateral femoral neck fracture with the femoral head displaced anteriorly (Figures 1(a) and 1(b)) and also an associated middle-third fracture of the shaft of the femur (Figure 1(c)). He also had an extra-articular distal femur fracture on the opposite side (AO type 3 3 A1). On arrival, his serum lactate level was 1.9 mmol/l indicating that he had been adequately resuscitated.

**Figure 2:**
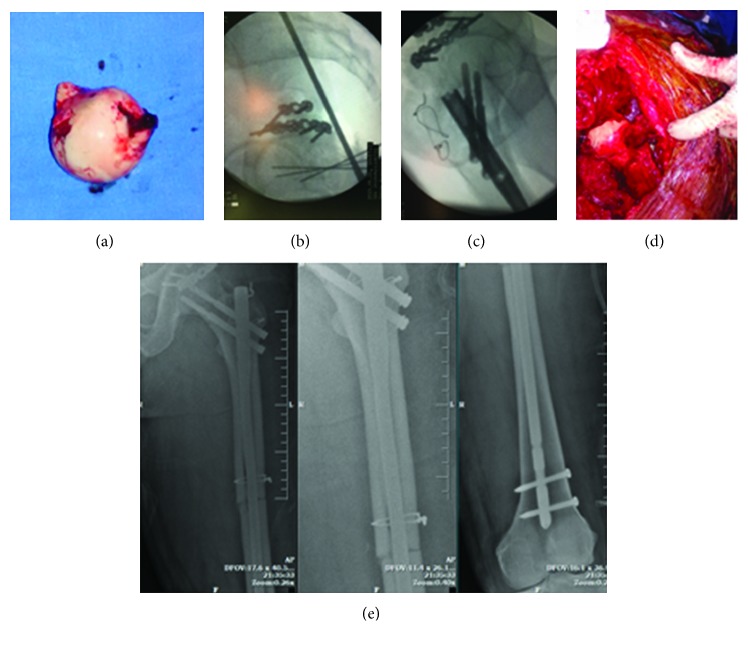
Intraoperative images of the displaced femoral head (a) which was reduced and fixed following the acetabulum fixation (b, c, d). Postoperative radiograph (e).

He was taken for definitive surgery 9 hours after arrival. The patient was positioned in the lateral position and a posterolateral approach was planned to address the acetabular and femoral head fractures. Upon dissection, the femoral head (Figure 2(a)) was found to have buttonholed and displaced anteriorly through the capsule which was found to be torn. The posterior wall of the acetabulum was addressed using two contoured reconstruction plates (Figure 2(b)). Using the trochanteric flip osteotomy, better access to the femoral neck was achieved and the femoral head was reduced anatomically and secured with K-wires. Then the femoral shaft fracture was reduced by opening the fracture site and held with a clamp. The femoral head fracture and the shaft fracture were fixed with an antegrade femoral nail with two screws securing the femoral head (Figures 2(c) and 2(d)). The flip osteotomy was fixed using a tension band wire and the joint was reduced. The torn capsule was sutured. Closure was done in layers. The operating time was 4 hours and the intraoperative blood loss was 600 ml. Three days following this surgery, the contralateral distal femur fracture was addressed using a titanium locking plate. The postoperative period was uneventful.

**Figure 3:**
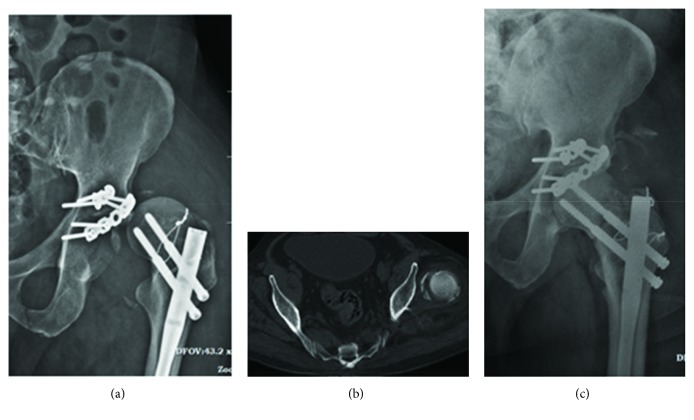
Anterior dislocation of the hip joint (a, b) following an episode of myoclonus which was managed by open reduction (c).

Three weeks after the initial surgeries, the patient experienced an episode of rhythmic contractions of the lower limbs at his home. He presented to us with an anterior dislocation of the left hip joint (Figures 3(a) and 3(b)). An open surgery was done to reduce the left hip joint (Figure 3(c)). Considering the displacement of the femoral head at the time of initial presentation, the chances of avascular necrosis of the femoral head was explained to the patient. The patient was also on follow-up treatment with a neurologist to manage the myoclonus problem.

**Figure 4:**
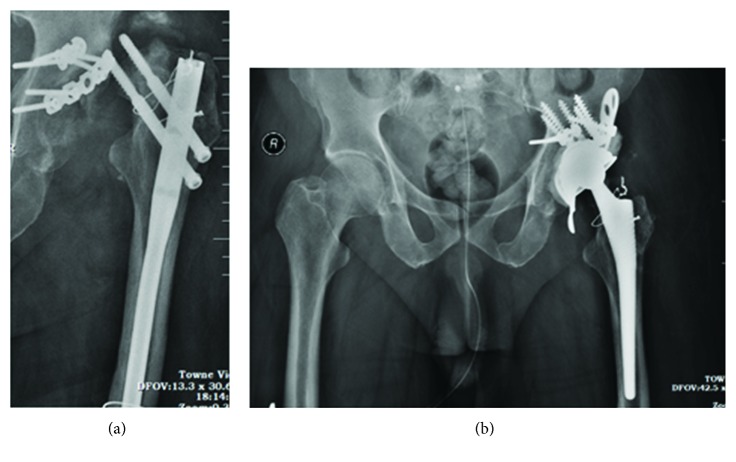
Avascular necrosis of the left femoral head (a) which was managed by implant removal and left total hip replacement (b) after union of the femur shaft fracture.

Four weeks after the surgery to relocate the femoral head, non-weight-bearing mobilization was initiated. As expected, avascular necrosis of the femoral head occurred (Figure 4(a)) and we waited for union of the femoral shaft to occur as any procedure to address the femoral head would require removal of the intramedullary nail.

Eight months after the initial surgery and after union of the femoral shaft fracture, the patient was planned for total hip replacement surgery. Through a posterior approach, the antegrade femoral nail was removed. The acetabulum was reconstructed using a cage, and an uncemented hydroxyapatite-coated stem was used for the femur (Figure 4(b)). A ceramic on a polybearing surface was used. Postoperatively, there was no shortening of the limb. Immediate full weight-bearing mobilization was started using walker support. One year following the hip replacement surgery and 22 months following the initial trauma, the patient was ambulatory without any support and able to do all activities with an LEFS (lower extremity functional score) of 72. The radiographs showed complete union of all the fractures and there was no loosening of the femoral prosthesis.

## 3. Discussion

Floating hip injuries are a rare presentation [1, 2, 4]. Moreover, a case with a similar presentation to the one discussed in our report is even more rare. To our knowledge, only two case reports of concomitant acetabular fracture with femoral head and femur shaft fracture have been published before. However, our case is unique with regard to the femoral head being extruded out and displaced out of the capsule. The management of such cases is difficult due to their low incidence and paucity in literature regarding their management [4–7].

Whenever one comes across a patient with the associated fractures as shown in our case, two main issues need to be planned before surgery. The first issue is with regard to which fracture will be addressed first, and the second issue is with regard to the implant to be used. Literature has shown that different surgeons have addressed different fractures in varying sequences. While Kregor [6] suggested prioritising the acetabular fracture first in fixation, Liebergall et al. [1] stated that fixing the femur first would help in easy reduction and traction while fixing the acetabulum. There has also been a disparity in the choice of implants between authors, with some authors suggesting a single implant and other authors suggesting separate implants for separate fractures [5–7]. We feel that these decisions have to be made based on a case-to-case basis.

In our case, since the femoral head and the acetabulum could be approached using a posterior approach, we employed it and since a single implant (antegrade femoral nail) would help in addressing all the fractures, we used it. Hence, we suggest that the management plan and the choice of implant for such complex injuries need to be planned from a case-to-case basis. The morphology and the complexity of the fracture influence the decision making, surgical approach, and the choice of implant.

Two case reports of similar type of injuries have been reported earlier. Duygulu et al. [8] managed their case with an antegrade nail for the femoral neck and shaft fractures followed by acetabular fixation, whereas Irifune et al. [9] employed screws to fix the femoral neck and a retrograde nail for the femur shaft fracture followed by the acetabular fixation. However, our case presented with a more complex fracture of the femoral neck which was different from the other two cases. The complexity of our injury was severe and such injuries have shown to be associated with a high chance of avascular necrosis (AVN) of the femoral head [10]. In our case, the AVN was addressed at the later stage with a total hip replacement. The plan of doing a total hip replacement at the initial stage was not possible in our case due to the concomitant fracture of the femur shaft.

To the best of our knowledge, such a complex case of a floating hip injury has not been reported. Though the chances of avascular necrosis was high in our case due to the fracture pattern of the femoral head, we decided to address the fracture with the same implant fixing the femur shaft as it was the best option for managing this fracture. Unfortunately, our patient had a hip dislocation following a myoclonus episode which was addressed appropriately by an open reduction of the joint. Eventually following AVN and after fracture union of the shaft, total hip replacement was done which led to a good functional outcome.

## 4. Conclusion

Ipsilateral acetabular fractures with femoral neck and femoral shaft fractures are very rare and a surgical challenge. Each case needs to be planned and addressed differently based on the complexity of the fracture, and our case report throws light on tackling this complex injury.
